# Applying GRADE-CERQual to qualitative evidence synthesis findings: introduction to the series

**DOI:** 10.1186/s13012-017-0688-3

**Published:** 2018-01-25

**Authors:** Simon Lewin, Andrew Booth, Claire Glenton, Heather Munthe-Kaas, Arash Rashidian, Megan Wainwright, Meghan A. Bohren, Özge Tunçalp, Christopher J. Colvin, Ruth Garside, Benedicte Carlsen, Etienne V. Langlois, Jane Noyes

**Affiliations:** 1 Norwegian Institute of Public Health, Oslo, Norway; 2 Health Systems Research Unit, South African Medical Research Council, Cape Town, South Africa; 3 School of Health & Related Research (ScHARR), University of Sheffield, Sheffield, UK; 4 Department of Health Management and Economics, School of Public Health, Tehran University of Medical Sciences, Tehran, Iran; 5 Information, Evidence and Research Department, Eastern Mediterranean Regional Office, World Health Organization, Cairo, Egypt; 6 Division of Social and Behavioural Sciences, School of Public Health and Family Medicine, University of Cape Town, Cape Town, South Africa; 7 UNDP/UNFPA/UNICEF/WHO/World Bank Special Programme of Research, Development and Research Training in Human Reproduction, Department of Reproductive Health and Research, WHO, Geneva, Switzerland; 8 European Centre for Environment and Human Health, University of Exeter Medical School, Exeter, UK; 9 Uni Research Rokkan Centre, Bergen, Norway; 10 Alliance for Health Policy and Systems Research, World Health Organization, Geneva, Switzerland; 11 School of Social Sciences, Bangor University, Bangor, UK

**Keywords:** Qualitative research, Qualitative evidence synthesis, Systematic review methodology, Research design, Methodology, Confidence, Guidance, Evidence-based practice, Recommendations for practice, GRADE

## Abstract

The GRADE-CERQual (‘Confidence in the Evidence from Reviews of Qualitative research’) approach provides guidance for assessing how much confidence to place in findings from systematic reviews of qualitative research (or qualitative evidence syntheses). The approach has been developed to support the use of findings from qualitative evidence syntheses in decision-making, including guideline development and policy formulation. Confidence in the evidence from qualitative evidence syntheses is an assessment of the extent to which a review finding is a reasonable representation of the phenomenon of interest. CERQual provides a systematic and transparent framework for assessing confidence in individual review findings, based on consideration of four components: (1) methodological limitations, (2) coherence, (3) adequacy of data, and (4) relevance. A fifth component, dissemination (or publication) bias, may also be important and is being explored. As with the GRADE (Grading of Recommendations Assessment, Development, and Evaluation) approach for effectiveness evidence, CERQual suggests summarising evidence in succinct, transparent, and informative Summary of Qualitative Findings tables. These tables are designed to communicate the review findings and the CERQual assessment of confidence in each finding. This article is the first of a seven-part series providing guidance on how to apply the CERQual approach. In this paper, we describe the rationale and conceptual basis for CERQual, the aims of the approach, how the approach was developed, and its main components. We also outline the purpose and structure of this series and discuss the growing role for qualitative evidence in decision-making. Papers 3, 4, 5, 6, and 7 in this series discuss each CERQual component, including the rationale for including the component in the approach, how the component is conceptualised, and how it should be assessed. Paper 2 discusses how to make an overall assessment of confidence in a review finding and how to create a Summary of Qualitative Findings table. The series is intended primarily for those undertaking qualitative evidence syntheses or using their findings in decision-making processes but is also relevant to guideline development agencies, primary qualitative researchers, and implementation scientists and practitioners.

**Electronic supplementary material:**

The online version of this article (10.1186/s13012-017-0688-3) contains supplementary material, which is available to authorized users.

## Why an approach to assessing confidence in the evidence from reviews of qualitative research is needed

Decisions on health, social care, and other interventions, programmes, and policies need to be based on the best available evidence [1]. While different stakeholders may attach different importance to different types of evidence [2, 3], there is a wide agreement that a broad range of evidence is needed to inform decisions. This is particularly so for more complex interventions or policies as well as for programmes or policies whose implementation may impact across institutions and systems, such as across schools or across the education, health, or social care system. For example, evidence may be needed on the values that people attach to different outcomes, on effects of an intervention on health or social outcomes, on the acceptability and feasibility of the intervention, on resource use and cost-effectiveness, on equity impacts, on ethics, and on implementation and scale-up considerations at different levels [1, 4, 5]. Diverse evidence may also be needed to understand why evidence-informed policies are not adopted in specific jurisdictions or are not implemented successfully [6–8]. This is an important consideration across all settings, but particularly in low- and middle-income countries where resources are limited and need to be used effectively [1, 9]. Data from qualitative research contributes critical information to addressing this need.

Qualitative research aims to explore people’s perceptions and experiences of the world around them, including their perspectives on health and illness, health and social care services, and wider health and social system policies and processes. In recent years, systematic reviews of qualitative research (also known as qualitative evidence syntheses) have become more common and the methods for undertaking these reviews are now well developed [10–12]. Evidence from qualitative evidence syntheses is increasingly incorporated into decision-making processes, including in health technology assessments, guideline development [13], and policy formulation, to complement evidence on the effects of interventions and on resource use. Qualitative evidence is also now being used within decision support tools such as the DECIDE evidence-to-decision frameworks [4] and SURE evidence-based policy briefs [14] and to inform decisions on implementation strategies. This wider use of qualitative evidence, including by organisations such as the World Health Organization (WHO), the European Commission Initiative on Breast Cancer and the National Institute for Health and Care Excellence (NICE) in the UK, has highlighted the need for the development of approaches that help users in deciding how much emphasis to give to such evidence in their decisions [15]. However, prior to the development of the approach described in this paper, there was no accepted, structured method for assessing confidence in the evidence from qualitative evidence syntheses [16]. The lack of such methods may constrain the use of qualitative evidence to inform decision-making.

The ‘Confidence in the Evidence from Reviews of Qualitative research’ (GRADE-CERQual) approach provides guidance for assessing how much confidence to place in findings from qualitative evidence syntheses. It complements other ‘Grading of Recommendations Assessment, Development, and Evaluation’ (GRADE) tools for assessing how much confidence to place in evidence on the effectiveness and harms of interventions and their resource use and in evidence about diagnostic tests [17, 18]. The guidance in this series has been developed in collaboration and agreement with the GRADE Working Group (www.gradeworkinggroup.org).

## Aims of the CERQual approach

The GRADE-CERQual approach (hereafter referred to as CERQual) has been developed to support people using findings from qualitative evidence syntheses in decision-making processes. CERQual allows the user to make a transparent assessment of how much confidence decision-makers and other users can place in individual review findings from syntheses of qualitative evidence. We define a review finding as an analytic output from a qualitative evidence synthesis that, based on data from primary studies, describes a phenomenon or an aspect of a phenomenon [16]. Many involved in using the findings of qualitative evidence syntheses may already be making these assessments of confidence intuitively or informally. As we see it, there are two main concerns with this: firstly, such assessments are not transparent and it is therefore not possible for others to see how the assessments were made and decide whether they agree with these decisions. Secondly, different assessors may use different criteria for assessing confidence and so assessments are not systematised across assessors (or even from one assessment to another, for the same assessor). Combined with the lack of transparency, this makes it difficult to understand, and where necessary critique, the basis for assessments. Broadly speaking, CERQual seeks to systematise the process of assessing confidence in the evidence from qualitative evidence syntheses and make these assessments explicit and transparent.

In developing CERQual, we were informed by the principles and methods of qualitative research and have also sought to apply lessons learned from the GRADE Working Group’s development of similar tools for other types of evidence. Table 1 lists the strengths of the CERQual approach, many of which are shared with other GRADE tools. CERQual is an emerging approach, and our knowledge of how to apply it is evolving. We therefore anticipate that guidance on applying CERQual will also evolve over time.

**Table 1:** Strengths of the CERQual approach

Strengths related to how the approach was developed:	
• Developed by a diverse group of international methodologists, qualitative researchers, systematic review authors and guideline developers. A few members of the group were also involved in health care decision making	
• Refined over several years through testing on a substantial number of qualitative evidence syntheses and through several rounds of consultations with academics and users in relevant fields	
Strengths related to the design of the approach:	
• Uses terminology, concepts and theoretical underpinnings that are sensitive to qualitative research	
• Provides explicit guidance on which concerns/threats to consider that may lead users to lower their confidence in the evidence	
• Makes judgements about confidence in qualitative evidence more transparent	
• The approach is independent of specific primary qualitative research methods and methods of synthesis	
• Assessments of confidence are based on multiple interdependent components	
Strengths related to the uses of the approach:	
• Assessments can be used within diverse decision making processes, including guideline development and health technology assessments, alongside GRADE assessments for other forms of evidence	
• The approach is congruent with other GRADE approaches for other types of evidence, and so can be easily integrated with these other approaches in decision making	
• The approach is well received and understood by stakeholders, when used in decision making processes including guideline development	
• Within decision making processes, CERQual may facilitate the use of qualitative evidence to address a range of issues. These include which outcomes are important to stakeholders; the acceptability and feasibility of interventions, including differences in views across different stakeholder groups; considerations regarding implementation; and the unintended consequences of interventions	

## Assumptions underlying the development of CERQual

As a pragmatic approach, CERQual makes several assumptions and acknowledgements regarding ongoing methodological debates in the field of qualitative research:

- We acknowledge that some within the qualitative research community have argued that synthesising data across multiple qualitative studies challenges the integrity of the contributing primary studies and that findings from this synthesis process may therefore not be trustworthy (e.g., [19–21]). However, in our approach to qualitative evidence synthesis and the development of CERQual, we have adopted the ‘subtle realist’ position [22] which maintains that the existence of phenomena does not depend on our subjective perceptions of them. In other words, social reality is not entirely constructed. Based on this, we suggest that synthesis can potentially provide a deeper understanding of a phenomenon than is achievable by any one single study, that this understanding can be viewed as trustworthy, and that it is therefore desirable to synthesise data from multiple qualitative studies. Others have made similar arguments in relation to ethnography, noting that comparisons of different ethnographic studies ‘are fruitful because they lead to empirical generalisations, they expose analytical problems, and they allow for falsification of hypotheses’ ([23] page 207).
- We acknowledge debates on the most appropriate methods for synthesis but argue that concerns regarding the synthesis process and its outputs should be addressed in *how* a synthesis is undertaken (for example, by using methods that help to preserve the context of the primary studies in the analysis process) rather than being seen as a complete barrier to conducting syntheses or to the practical usefulness of qualitative synthesis findings to decision-makers [24].
- CERQual assumes that qualitative research, in addition to its more interpretative and exploratory functions, has an instrumental role to play in informing decisions. In other words, qualitative research holds the potential to produce knowledge that can directly inform decision-making processes.
- CERQual acknowledges that a well-conducted qualitative evidence synthesis does not automatically produce useful findings applicable to a range of contexts. As with primary qualitative research, sophisticated processes of analysis and interpretation are required. CERQual aims to accommodate the interpretivist nature of qualitative synthesis by, for example, encouraging the review authors to examine possible theoretical contributions and to be sensitive to the importance of context when assessing confidence in the evidence
- The CERQual approach is intended to be applied to well-conducted syntheses that report their methods and limitations in a transparent way. We believe that applying CERQual to the findings of a poorly conducted or poorly reported synthesis would be challenging and would not yield useful results. Paper 2 in the series provides guidance on assessing how well a review was conducted [25].

Additional file 1 describes the purpose of CERQual and what it is not intended to address.

## How was the CERQual approach developed?

Overall, we used a pragmatic and iterative approach to develop each CERQual component by brainstorming concepts within the development team, undertaking formal and informal searching of the literature for definitions, following up relevant citations, talking to experts in the field of qualitative evidence synthesis, developing consensus through multiple face-to-face meetings and teleconferences, and seeking feedback from ongoing engagement with the qualitative evidence synthesis community, the GRADE Working Group and organisations that commission, produce, or use systematic reviews.

### Initial development of the CERQual approach

The initial stages of the process for developing CERQual, which started in 2010, are outlined elsewhere [16] (see Additional file 2). This work led to an approach in which four components—methodological limitations, coherence, adequacy of data, and relevance—contribute to an overall assessment of confidence in an individual review finding. We presented this version of the approach in 2015 to a group of 27 invited methodologists, researchers, and end users from more than 12 international organisations and 10 countries, with a broad range of experience in qualitative research, the development of GRADE, or guideline development. This group, together with others who registered interest in the approach, constitutes a wider CERQual Project Group and played a significant role in the refinement of the approach.

### Further development of the CERQual approach

We took several other steps to further develop the approach. Firstly, we undertook a coordinated programme of implementation activity involving training workshops, seminars, and presentations during which we actively sought, collated, and shared feedback to enhance understanding and further development of the CERQual components and their practical application. Between 2015 and 2017, at least 10 workshops and seminars and eight presentations were undertaken. Secondly, in 2015 and 2016, we implemented a small-group feedback approach in which we facilitated brief discussions of individual CERQual components, either within our host organisations or in response to specific invitations from other organisations. Thirdly, we applied the CERQual approach within diverse qualitative evidence syntheses in the areas of health and social care [6–8, 26–33] and also supported other teams in using CERQual by providing guidance through face-to-face or virtual training meetings and commenting on draft Summaries of Qualitative Findings tables. At least 10 syntheses were supported in this way (for example, [34, 35]). We then gathered structured feedback from early users of CERQual through an online feedback form that was made available to all CERQual users and through short individual discussions with six members of review teams and two members of the CERQual Project Group. The questions included in the online feedback form and individual discussions are available in Additional file 3. These experiences and the feedback we received from users contributed to the further refinement of the approach, including how each component should be conceptualised and applied. As far as possible, we used a consensus approach in these processes. While no formal guidelines exist for the development of an assessment approach of this kind, our process closely resembles the recommended approach for developing guidelines for reporting research processes [36].

### The role of dissemination bias

In earlier discussions of CERQual, we identified dissemination bias (sometimes called publication bias) as a potential fifth component in assessing how much confidence to place in qualitative evidence synthesis findings. We initiated pilot work to explore how dissemination bias has been conceptualised in qualitative research, its perceived scope, and how it might be conceptualised. Some of this work is discussed in paper 7 in this series [37] and elsewhere [38, 39].

## An overview of the CERQual approach to assessing confidence in the evidence

We have defined confidence in the evidence as an assessment of the extent to which a review finding is a reasonable representation of the phenomenon of interest. This assessment communicates the extent to which the review finding is likely to be substantially different from the phenomenon of interest. By ‘substantially different’, we mean different enough that it might change how the finding influences a decision about health, social care, or other interventions [16]. For instance, if a review finding suggests that a new social care intervention is very acceptable to most service users and we have high confidence in this finding (indicating that it is highly likely that the finding is a reasonable representation of acceptability to service users), decision-makers may assess that it is appropriate to recommend that the intervention be implemented, assuming that the desirable effects outweigh the undesirable effects for other decision criteria. However, if we have very low confidence in this finding and it is therefore unclear whether the intervention is acceptable to most service users, decision-makers may assess that it is not appropriate to recommend its implementation.

**Fig. 1:**
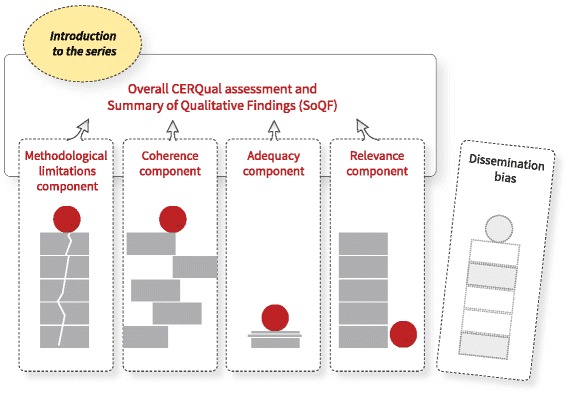
Overview of the GRADE-CERQual series of papers

CERQual involves an assessment of each individual review finding in terms of four components: (1) methodological limitations, (2) coherence, (3) adequacy of data, and (4) relevance (Table 2). The assessments of the four components collectively contribute to an overall assessment of whether findings from a qualitative evidence synthesis provide a reasonable representation of the health or social care issue, intervention, or programme (phenomenon) of interest (Fig. 1). Our approach is comparable to that for GRADE for effectiveness where review authors assess the confidence, or certainty, in the estimates of effect for each critical and important outcome by evaluating risk of bias, directness, inconsistency, imprecision, and publication bias [17].

**Table 2:** Definitions of the components of the CERQual approach

Component	Definition
Methodological limitations	The extent to which there are concerns about the design or conduct of the primary studies that contributed evidence to an individual review finding
Coherence	An assessment of how clear and cogent the fit is between the data from the primary studies and a review finding that synthesises that data. By ‘cogent’, we mean well supported or compelling
Adequacy of data	An overall determination of the degree of richness and quantity of data supporting a review finding
Relevance	The extent to which the body of evidence from the primary studies supporting a review finding is applicable to the context (perspective or population, phenomenon of interest, setting) specified in the review question

When using CERQual, we assess confidence in each individual review finding. We acknowledge that review findings can be presented in a range of ways (e.g., as themes or theories) as well as at different levels (e.g., descriptive/aggregative and interpretive). We report confidence in each review finding as either high, moderate, low, or very low confidence (Table 3).

**Table 3:** Descriptions of level of confidence in a review finding in the CERQual approach [16]

Level	Definition
High confidence	It is highly likely that the review finding is a reasonable representation of the phenomenon of interest
Moderate confidence	It is likely that the review finding is a reasonable representation of the phenomenon of interest
Low confidence	It is possible that the review finding is a reasonable representation of the phenomenon of interest
Very low confidence	It is not clear whether the review finding is a reasonable representation of the phenomenon of interest

A key product of a CERQual assessment, as with other GRADE approaches, is a succinct, transparent, and informative Summary of Qualitative Findings (SoQF) table. The SoQF table facilitates the use of review findings in decision-making processes and is purpose-designed to communicate to users both the overall confidence assessment for each review finding and an explanation of this assessment. In paper 2 in this series, we describe how synthesis authors proceed from a full review finding to a summary of a review finding, for inclusion in a SoQF table [25]. The SoQF table is complemented by a CERQual Evidence Profile which includes the explicit judgements for each CERQual component that contributes to the overall confidence assessment for each review finding [25]. Additional file 4 outlines minimum criteria that need to be met for review authors to assert fidelity to the GRADE-CERQual approach. We noted earlier that the development of CERQual has been informed by the principles and methods underlying both primary qualitative research and qualitative evidence synthesis. Those applying CERQual should, in our experience, have a good understanding of both qualitative primary research methods and qualitative evidence synthesis methods to apply the approach appropriately.

As noted in relation to GRADE for evidence of effectiveness, confidence in the evidence exists on a continuum. One limitation of a CERQual assessment is the discrete categorisation of confidence into levels (high, moderate, etc.), which inevitably involves a degree of arbitrariness. However, we would argue that the accessibility and transparency of this approach outweigh its limitations [40].

## Applying CERQual across types of qualitative data and synthesis methods

An ever-expanding array of qualitative synthesis methods are available [41, 42]. Our aspiration is that CERQual could be applied to findings from syntheses based on any type of qualitative data that use a variety of synthesis methods and that address a range of questions. Within the domains of health and social care, this includes questions such as people’s views or experiences of a health or social care issue, how different stakeholders and population groups value different health or social care outcomes, stakeholders’ views on the acceptability and feasibility of health or social care interventions or options and on how an intervention might work, and factors affecting the implementation of an intervention or option. So far, experience in using CERQual has been concentrated in reviews with findings that are aggregative in nature, for example, related to health care users’ and providers’ experiences and understanding of health issues and health service delivery [6, 7, 28, 29, 31]. We have yet to gather experience about the use of CERQual on the full scope of synthesis methods and types of review findings. This is an important area for future research. For example, we need to explore how decision-makers use review findings at different levels of abstraction: are findings that carry a high level of abstraction as immediately useful to decision-makers as those grounded within contextual parameters such as time, place, and culture? We also need to explore the usefulness for decision-making of findings from syntheses that use different synthesis methods.

## Purpose and structure of this series of papers

**Fig. 2:**
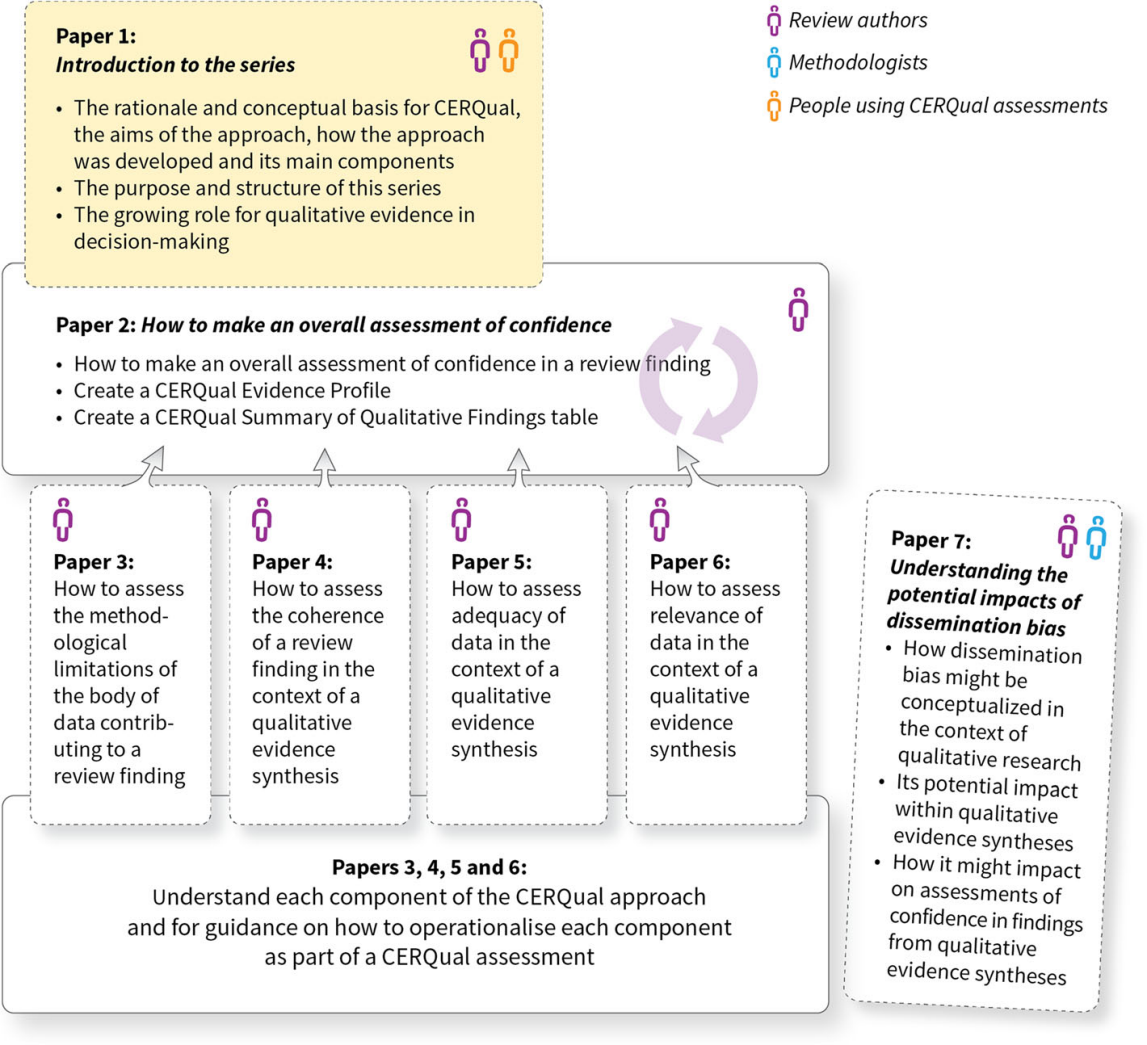
How the papers in the GRADE-CERQual series can be used

This series of papers aims to provide guidance to users on *how to apply* the GRADE-CERQual approach. The series takes the reader through all the stages involved in making an assessment of confidence in findings from qualitative evidence syntheses, including how we have conceptualised each component of CERQual and how these components relate to other concepts in the fields of primary qualitative research and qualitative evidence synthesis. Figure 1 provides an overview of the series and the CERQual approach, while Fig. 2 provides a guide for navigating through the series, including an overview of the purpose of each paper and the relevance of each paper to review authors, methodologists, and people using CERQual assessments.

The second paper in the series discusses how to move from a full description of a review finding to a summary of a review finding—an important step in the process of applying CERQual; how to determine to which review findings to apply CERQual; how to make an overall CERQual assessment; and how to create a Summary of Qualitative Findings table [25]. The next four papers in the series describe each of the four CERQual components in depth, including how this component is conceptualised and how it should be operationalised as part of a CERQual assessment [43–46]. The final paper in the series discusses dissemination bias in qualitative research and its potential implications for qualitative evidence synthesis and CERQual assessments [37].

This series is the first to discuss in detail how to apply the GRADE-CERQual approach. The series has been developed primarily for those undertaking qualitative evidence syntheses or those supporting the use of the findings of such syntheses in decision-making processes. The series is also relevant to guideline development and health technology assessment agencies, decision-makers, and qualitative researchers. It will also be useful for those seeking to understand recommendations and other decisions to which qualitative evidence with CERQual assessments have contributed. We will provide further guidance on using CERQual within decision-making processes, including in guideline development and in additional papers planned within our publications strategy.

Different readers will use this series in different ways (Fig. 2). Those conducting qualitative evidence syntheses may choose to read the series in its entirety to help them to apply the approach. Those supporting the use of qualitative evidence in decision-making may use the series as a reference guide to better understand how CERQual assessments are undertaken and how they are to be interpreted. Qualitative researchers may use the articles to understand the diverse information to be reported in primary qualitative studies, so as to facilitate the application of CERQual to future syntheses. Implementation researchers can use these articles in undertaking qualitative evidence syntheses related to implementation and in using the findings of these syntheses.

In writing this series, we have tried to draw on examples from published or ongoing syntheses addressing a disparate range of questions and contexts in order to show how CERQual should be applied. We also highlight key methodological issues to be considered at each stage or that arise from using CERQual. However, because CERQual is a relatively new approach, the pool of worked examples is not yet extensive and is drawn largely from the areas of health and social care. We believe, though, that CERQual can and should be applied to findings from qualitative evidence syntheses across all fields, including agriculture, crime and justice, education, the environment and international development. We encourage readers to share with us their applied examples from these domains.

## Conclusions: the widening influence of qualitative evidence

The increasing use of qualitative evidence within a range of decision-making processes, and the growing awareness of the roles that qualitative evidence can play in decision-making [47], suggests that we are entering a new era for qualitative research. Perhaps the most important function that qualitative evidence can play in decision-making, including in the development of guidelines and health technology assessments, is to represent the views and experiences of a wide range of stakeholders. Engaging key stakeholders, such as the public and health care consumers, providers, and managers, in decision-making is widely promoted and recognised as a key to encouraging participative democracy and public accountability [13, 48–54]. However, to date, stakeholder engagement in decision-making in many settings is largely accomplished through dialogues and consultations with these stakeholders [55] and through including stakeholder representatives in decision-making forums, such as guideline panels [56]. While important and valuable, such engagement is limited by the knowledge and experience that individuals can bring to such dialogues and forums. For decisions that may impact on very large numbers of people, individual stakeholder representatives alone cannot be expected to represent effectively the views of all affected groups and consultations seldom reach all sectors of society. By drawing on the global qualitative research literature, qualitative evidence syntheses have the potential to greatly widen the range of views and experiences represented in decision-making, thereby helping to ensure that the choices made take these views into account. This may also contribute to increased transparency and accountability in public decision-making [57, 58]. CERQual plays a key role in this process by providing decision-makers with assessments of how much confidence they can place in such evidence.

Many decision-makers acknowledge the need to widen the range of evidence that they examine, to address questions such as the acceptability of interventions and programmes as well as other factors that might impact on their implementation. Many are aware that qualitative research provides a valuable pool of knowledge from which they can draw. We believe that the CERQual approach will help to increase the usability of findings from qualitative evidence syntheses, including use by those who are implementing interventions across fields such as education, health, social care and justice. CERQual will evolve as we gain more experience in applying the approach across diverse review findings derived from different synthesis approaches. Table 4 identifies several important areas for further methodological research, including how to apply CERQual in syntheses that include qualitative and quantitative data; how best to present CERQual assessments together with other kinds of evidence; ways of applying CERQual to syntheses of sources that have not used formal qualitative research procedures; and whether CERQual requires adaptation for application to more interpretive synthesis outputs, such as logic models. We hope that those using the approach will help us to develop and improve what is presented in this series. We encourage readers to join the CERQual Project Group and to engage with our website (www.cerqual.org), on which new developments will continue to be flagged.

**Table 4:** Way forward and research agenda for CERQual

The following steps are needed to further develop the approach:
• To date, there is little collective experience of applying CERQual in the context of mixed method syntheses that include qualitative and quantitative data. Whether the approach needs to be adapted for this context needs to be explored. An important concern is whether assessing the quantitative and qualitative elements of a mixed-methods study individually, using separate approaches, risks under-valuing the contribution of review findings based on integrated data
• In some decision making processes, CERQual assessments of qualitative evidence may be presented alongside other GRADE assessments for data on intervention effectiveness and resource use.User testing is needed to explore how best to present this range of assessments to evidence users
• Our aim is that CERQual can be applied to review findings based on any kind of qualitative data. However, we do not have experience of applying the approach to syntheses where the primary material includes sources that are textual in nature but are not the output of formal qualitative research procedures. Such sources include blogs, online discussion group transcripts or newspaper reports. Further work is needed to examine how the approach can be used for such data
• We need to gather experience and, if necessary, adapt CERQual for syntheses of primary studies outside the field of health and health care research
• We need further work on whether CERQual needs to be adapted for application to more interpretive outputs from syntheses, such as logic models and findings from synthesis methods such as meta-ethnography

## Open peer review

Peer review reports for this article are available in Additional file 5.

## Additional files

Additional file 1: The purpose of CERQual and what CERQual is not intended to address. (PDF 621 kb)

Additional file 2: Methods used to develop the CERQual approach—2010 to 2015. (PDF 647 kb)

Additional file 3: Questions included in the CERQual online feedback form and short individual discussions. (PDF 468 kb)

Additional file 4: Minimum criteria for fidelity to the GRADE-CERQual approach in a qualitative evidence synthesis. (PDF 353 kb)

Additional file 5: Open peer review reports. (PDF 142 kb)

## Abbreviations

CERQual: Confidence in the Evidence from Reviews of Qualitative research
DECIDE: Developing and Evaluating Communication Strategies to Support Informed Decisions and Practice Based on Evidence (an EU-funded research project)
GRADE: Grading of Recommendations Assessment, Development, and Evaluation
SoQF: Summary of Qualitative Findings
SURE: Supporting the use of research evidence for policy in African health systems (an EU-funded research project)
