# Supplementary material for: Applying GRADE-CERQual to qualitative evidence synthesis findings: introduction to the series
Source: Implement Sci. 2018 Jan 25;13(Suppl 1):2. doi: 10.1186/s13012-017-0688-3 (PMC5791040; doi:10.1186/s13012-017-0688-3)
Supplement: Supplementary file 1 — The purpose of CERQual and what CERQual is not intended to address. (PDF 621 kb) [file 13012_2017_688_MOESM1_ESM.pdf]

Additional file 1: The purpose of CERQual and what CERQual is not intended to address*

The CERQual approach transparently assesses and describes how much confidence to place in individual review findings from syntheses of qualitative evidence.<br><br>CERQual is not intended as a tool for the following purposes:<br>• Critical appraisal of the methodological limitations of an individual qualitative study<br>• Critical appraisal of the methodological limitations of a qualitative evidence synthesis (i.e. how well a qualitative evidence synthesis was conducted)[#]<br>• Appraisal of quantitative or mixed methods data or quantitative studies of quality of care or quality of life<br>• Assessing how much confidence to place in the findings from what are sometimes described as “narrative” or “qualitative” summaries of the effectiveness of an intervention, in systematic reviews of effectiveness where meta-analysis is not possible<br>• Assessing how much confidence to place in the overall findings of a qualitative evidence synthesis or providing a composite assessment of all of the findings of a review. Rather, it focuses on assessing how much confidence to place in individual review findings from qualitative evidence syntheses

* Adapted from [1]

[#] Note that an assessment of methodological limitations forms part of the CERQual approach for assessing confidence in an individual review finding [2].

References

1. Lewin S, Glenton C, Munthe-Kaas H, Carlsen B, Colvin CJ, Gulmezoglu M, Noyes J, Booth A, Garside R, Rashidian A: **Using Qualitative Evidence in Decision Making for Health and Social Interventions: An Approach to Assess Confidence in Findings from Qualitative Evidence Syntheses (GRADE-CERQual)**. *PLoS Med* 2015, **12**(10):e1001895.
2. Munthe-Kaas HM, Bohren M, Carlsen B, Glenton C, Lewin S, Colvin CJ, Tuncalp Ö, Noyes J, Booth A, Garside R *et al*: **Applying GRADE-CERQual to qualitative evidence synthesis findings - paper 3 of 7: how to assess methodological limitations**. *Implementation Science* 2017, **12 Suppl 2.**
