# Supplementary material for: Applying GRADE-CERQual to qualitative evidence synthesis findings: introduction to the series
Source: Implement Sci. 2018 Jan 25;13(Suppl 1):2. doi: 10.1186/s13012-017-0688-3 (PMC5791040; doi:10.1186/s13012-017-0688-3)
Supplement: Supplementary file 2 — Methods used to develop the CERQual approach—2010 to 2015. (PDF 647 kb) [file 13012_2017_688_MOESM2_ESM.pdf]

Additional file 2: Methods used to develop the CERQual approach – 2010 to 2015

The CERQual approach was initially developed in 2010 to support a panel that was using qualitative evidence syntheses to develop a new World Health Organization (WHO) guideline [1]. The technical team for this guideline needed an approach for consistently and transparently assessing and presenting any concerns about the qualitative evidence synthesis findings being used by the panel to inform the guideline.

To develop CERQual, we established a working group of researchers involved in undertaking evidence syntheses. We needed an approach that could be applied to data from common types of qualitative study designs (e.g., ethnography, case studies) and data (e.g., from interviews, observational), was easy to use, provided a systematic approach to making judgements, allowed these judgements to be reported transparently, and allowed judgements to be understood easily, including by readers without an in-depth understanding of qualitative methods. This work was informed by principles of qualitative research and by the principles used to develop GRADE for effectiveness [2].

CERQual was developed iteratively. Our first version included two components—methodological limitations and coherence—and was piloted on five syntheses [3-7]. In 2013, we presented the CERQual approach to researchers, methodologists, and decision makers at numerous events, including the Cochrane Colloquium [8] and a GRADE Working Group meeting. We revised the approach, based on feedback from these sessions, to include an additional two components. This expanded the approach to include four components: methodological limitations, coherence, adequacy of data, and relevance. We also identified a further potential factor —dissemination bias—as important but requiring further methodological research before it could be included in the CERQual approach.

To obtain further feedback, we presented the four-component version of the approach in 2014 to a group of 25 invited methodologists, researchers, and end users from more than twelve international organizations, with a broad range of experience in qualitative research, the development of GRADE, or guideline development.

(This text is adapted from [9])

References

1. WHO: **Optimizing health worker roles to improve access to key maternal and newborn health interventions through task shifting**. Geneva: World Health Organization; 2012.
2. Guyatt GH, Oxman AD, Vist GE, Kunz R, Falck-Ytter Y, Alonso-Coello P, Schunemann HJ, Group GW: **GRADE: an emerging consensus on rating quality of evidence and strength of recommendations**. *BMJ,* 2008, **336**(7650):924-926.
3. Bohren MA, Hunter EC, Munthe-Kaas HM, Souza JP, Vogel JP, Gulmezoglu AM: **Facilitators and barriers to facility-based delivery in low- and middle-income countries: a qualitative evidence synthesis**. *Reproductive health* 2014, **11**(1):71.

4. Colvin CJ, de Heer J, Winterton L, Mellenkamp M, Glenton C, Noyes J, Lewin S, Rashidian A: **A systematic review of qualitative evidence on barriers and facilitators to the implementation of task-shifting in midwifery services**. *Midwifery* 2013, **29**(10):1211-1221.
5. Glenton C, Colvin CJ, Carlsen B, Swartz A, Lewin S, Noyes J, Rashidian A: **Barriers and facilitators to the implementation of lay health worker programmes to improve access to maternal and child health: qualitative evidence synthesis**. *The Cochrane database of systematic reviews* 2013, **10**:CD010414.
6. Munthe-Kaas HM, Hammerstrøm KT, et al.: **Effekt av og erfaringer med kontinuitetsfremmende tiltak i barnevernsinstitusjoner**. Oslo: Norwegian Knowledge Centre for the Health Services; 2013.
7. Rashidian A, Shakibazadeh E, Karimi- Shahanjarini A, Glenton C, Noyes J, Lewin S, Colvin C, Laurant M: **Barriers and facilitators to the implementation of doctor-nurse substitution strategies in primary care: qualitative evidence synthesis (Protocol)**. *Cochrane Database of Systematic Reviews* 2013, **2**:CD010412.
8. Lewin S, Glenton C, Munthe-Kaas H, Carlsen B, Colvin C, Noyes J, Rashidian A: **Assessing how much certainty to place in findings from qualitative evidence syntheses: the CerQual approach.** Oral presentation, *20th Cochrane Colloquium*, Quebec 2013.
9. Lewin S, Glenton C, Munthe-Kaas H, Carlsen B, Colvin CJ, Gulmezoglu M, Noyes J, Booth A, Garside R, Rashidian A: **Using qualitative evidence in decision making for health and social interventions: an approach to assess confidence in findings from qualitative evidence syntheses (GRADE-CERQual)**. *PLoS Med* 2015, **12**(10):e1001895.
