# Supplementary material for: Applying GRADE-CERQual to qualitative evidence synthesis findings: introduction to the series
Source: Implement Sci. 2018 Jan 25;13(Suppl 1):2. doi: 10.1186/s13012-017-0688-3 (PMC5791040; doi:10.1186/s13012-017-0688-3)
Supplement: Supplementary file 3 — Questions included in the CERQual online feedback form and short individual discussions. (PDF 468 kb) [file 13012_2017_688_MOESM3_ESM.pdf]

Additional file 3: Questions included in the CERQual online feedback form and short individual discussions

What is your name?

What is your institution?

What is your email address?

What was the name of the review to which you applied GRADE-CERQual?

Please list all the names of those who used GRADE-CERQual (including yourself)

Was your team applying GRADE-CERQual to their own review or to someone's else's?

If the review is published please provide the reference here:

When did you carry out this work?

Was the review commissioned? (If yes, please indicate who commissioned it)

How did you hear about GRADE-CERQual?

Please indicate which, if any of the following sources of support you accessed while applying GRADE-CERQual:

- Website
- Publications
- CERQual Dropbox folder
- Direct contact with a member of the coordinating team
- Materials received at a seminar or training workshop
- Other:

Please describe your overall experience of applying GRADE-CERQual

What did you find challenging about the GRADE-CERQual approach?

Please describe any examples that exemplify a challenge you faced in assessing the "methodological limitations" component:

Please describe any examples that exemplify a challenge you faced in assessing the "relevance" component:

Please describe any examples that exemplify a challenge you faced in assessing the "adequacy of data" component:

Please describe any examples that exemplify a challenge you faced in assessing the "coherence" component:

Please describe any examples that exemplify a challenge you faced in making an overall assessment of confidence:

Could you have benefitted from additional support? If so, what kinds of support would be useful?

Please share any other feedback regarding your experience of using GRADE-CERQual

What (if applicable) were the reactions of stakeholders/decision-makers to your review findings and the GRADE-CERQual assessments?
