# Supplementary material for: Applying GRADE-CERQual to qualitative evidence synthesis findings: introduction to the series
Source: Implement Sci. 2018 Jan 25;13(Suppl 1):2. doi: 10.1186/s13012-017-0688-3 (PMC5791040; doi:10.1186/s13012-017-0688-3)
Supplement: Supplementary file 4 — Minimum criteria for fidelity to the GRADE-CERQual approach in a qualitative evidence synthesis. (PDF 353 kb) [file 13012_2017_688_MOESM4_ESM.pdf]

Additional file 4: Minimum criteria for fidelity to the GRADE-CERQual approach in a qualitative evidence synthesis

In order to assert fidelity to the GRADE-CERQual approach in a qualitative evidence synthesis (sometimes called a systematic review of qualitative studies), you need to meet the following minimum criteria:

1. Define “confidence in the evidence” consistently.
2. Give explicit consideration in your assessments of confidence to each of the GRADE-CERQual components: methodological limitations, coherence, adequacy and relevance.
3. Assess confidence in the evidence for each synthesis finding and express this using four categories: high, moderate, low and very low confidence.
4. Use the definitions developed by the GRADE-CERQual Project Group and approved by GRADE for the following elements of the CERQual approach:
    - Confidence in the evidence
    - The GRADE-CERQual components: methodological limitations, coherence, adequacy, relevance
    - Categories for confidence in the evidence: high, moderate, low, very low
5. Use a CERQual Qualitative Evidence Profile or Summary of Qualitative Findings Table to summarise the judgements for each synthesis finding in relation to each of the four CERQual components and the overall CERQual assessment of confidence[1]. In particular, describe transparently the reasons for grading down the confidence in the evidence for each synthesis finding and ensure that each finding, and accompanying CERQual assessment, can be traced back to the individual studies that support it.

[1] Some journals and other dissemination platforms may not be willing to publish a full CERQual Qualitative Evidence Profile and / or Summary of Qualitative Findings Table as part of the paper reporting the findings of a qualitative evidence synthesis. In such cases, these tables can be made available as supplementary materials to the paper or online elsewhere, with a link in the paper.
