# Supplementary material for: Applying GRADE-CERQual to qualitative evidence synthesis findings: introduction to the series
Source: Implement Sci. 2018 Jan 25;13(Suppl 1):2. doi: 10.1186/s13012-017-0688-3 (PMC5791040; doi:10.1186/s13012-017-0688-3)
Supplement: Supplementary file 5 — Open peer review reports. (PDF 142 kb) [file 13012_2017_688_MOESM5_ESM.pdf]

Open Peer Review reports

Original Submission		
25 Oct 2016	Submitted	Original Manuscript
20 Jan 2017	Reviewed	Reviewer Report - Elizabeth Nye

'Why an approach to assessing confidence in the evidence from reviews of qualitative research is needed'
* This section serves as a good, brief introduction to qualitative research, qualitative evidence synthesis, and use of qualitative research in decision-making.
* This section does not yet really answer the question posed in the heading - why an approach to assessing confidence is needed. I would have expected some discussion about a current lack of any structured/widely accepted approach to assess confidence in findings from these reviews and the implications of this gap as a way to set up the need for the CERQual approach.

'Aims of the CERQual approach'
* To what end would systematising and making these assessments of confidence more explicit improve this area? Is there something wrong with intuitively or informally assessing confidence of findings? What is the problem that producing the CERQual approach will address? Is it hoped that this approach will improve the quality of reviews and their reporting (i.e., the research side of things) or will it improve users' assessments of the findings (i.e., the user/decision-making side of things)? Or both?

'Assumptions underlying the development of CERQual'
* This is a very helpful section and clearly/succinctly outlines the authors' assumptions for the reader.

'Applying CERQual across types of qualitative data and synthesis methods'
* This section raises some very interesting questions for future research.

'How was the CERQual approach developed?'
* This section describes a comprehensive process of developing CERQual with many types of users and refers the reader elsewhere for additional details, if desired. Including this section is very helpful in this introductory paper.

'An overview of the CERQual approach to assessing confidence in the evidence'
* It would be helpful to add a short example to more concretely describe what is meant by '[…] different enough that it might change how the finding influences a decision about health, social care, or other interventions' (p. 10, including lns. 24-34). Grounding this explanation in a short but material example (real or hypothetical) would help readers gain a more tangible understanding.
* It is recommended that 'at least one member of the team applying CERQual has experience in both primary qualitative research and qualitative evidence synthesis' (p. 11, lns 33-36). Further explanation is needed. Why is this recommended? Is there research to demonstrate that users with experience in only one of these areas are insufficiently prepared to apply this approach? Would it have to be one member who has experience in both areas or could it involve two members who each have experience in one area? Also, and perhaps most importantly, what exactly is meant by 'experience' - this seems vague and

highly subjective. Finally, should readers interpret this recommendation to mean that if a group of users does not have a member with experience in both of these areas, then that group should not apply the CERQual approach to a review of qualitative research (or that if they do, the result will be of a lower quality)?

'Purpose and structure of this series of papers'
* This section provides a helpful overview of the series.

30 Jan 2017	Reviewed	Reviewer Report - Helen Smith

General comments
Thank you for the opportunity to review this paper. As a researcher who has published several qualitative evidence syntheses, I have heard of CERQual over the last few years but have never seen a formal description of the approach. With an increase in both the production and use of qualitative evidence syntheses across many areas of heath and healthcare, this series is timely. As the authors point out, the articles will potentially be of interest to a range of readers/ users. My comments are minor and mainly relate to making the concept accessible to a wide readership.
Specific comments
1. Page 5 line 5: Although the authors do mention it later on in the article, I think the fact that CERQual has been developed within health and healthcare (and that this is the context in which the article is written) and it has not yet been applied to other fields needs to be made clear at the beginning.
2. Page 5 line 15: related to the above comment, the reference to institutions and systems is a bit abstract and needs context to make it meaningful; are the authors really referring to health systems and institutions?
3. Page 6 line 44: Again the authors do define this later on, but the term 'individual review finding' is first used on page 6 and to avoid confusion for readers unfamiliar with this, perhaps it warrants a definition here? The value of this series of papers partly lies in the authors' ability to make the concept accessible to a wide readership, many of whom will not know what an individual review finding is.
4. Page 7 line 1: Table 1 summarises strengths of the approach in a long list. Could the authors categorise these strengths somehow? I.e. strengths related to how the tool has been developed, those that relate to the process/conduct and those that relate to its various uses.
5. Page 8 line 19: the applicability of CERQual to reviews that address different questions is discussed here. To make it really clear to a wide readership, the authors could provide further examples of the types of questions addressed by QES.
6. Pages 8-9: the process of developing CERQual, described here, seems to have been largely organic and unsystematic. Sub-headings might help the reader navigate this section better. Also its not particularly clear what a 'coffee CERQual' is, or why/how it is innovative.
7. Page 11 line 2: is CERQual applied in the same way, and indeed able to be applied, to the range of review findings mentioned here? i.e. are all four components equally applicable to a theory and a theme?
8. Page 13: the authors could elaborate here - especially for readers outside of the health field - is there any reason why CERQual couldn't be applied to reviews conducted in the fields of education, environment, agriculture etc?
9. Page 14: the reader must accept the authors' word that the approach has been well received, unless these experiences have been documented or written up elsewhere?

23 Jul 2017	Author responded	Author comments - Simon Lewin

Peer reviewer comments	Responses
**Reviewer #1:**	
'Why an approach to assessing confidence in the evidence from reviews of qualitative research is needed'<br>* This section serves as a good, brief introduction to qualitative research, qualitative evidence synthesis, and use of qualitative research in decision-making.	Thank you
* This section does not yet really answer the question posed in the heading - why an approach to assessing confidence is needed. I would have expected some discussion about a current lack of any structured/widely accepted approach to assess confidence in findings from these reviews and the implications of this gap as a way to set up the need for the CERQual approach.	On pages 5-6 of the paper, we provide a rationale for developing an approach to assessing confidence in the reviews from reviews of qualitative research. To further address the reviewer's point, we have added two sentences to this paragraph, and also cited our earlier paper on the CERQual approach that describes the rationale for the approach in more detail. The revised paragraph reads as follows:<br><br>"Qualitative research aims to explore people's perceptions and experiences of the world around them, including their perspectives on health and illness, health and social care services, and wider health and social system policies and processes. In recent years, systematic reviews of qualitative research (also known as qualitative evidence syntheses) have become more common and the methods for undertaking these reviews are now well developed [10-12] Evidence from qualitative evidence syntheses is increasingly incorporated into decision-making processes, including in health technology assessments, guideline development [13] and policy formulation, to complement evidence on the effects of interventions and on resource use. Qualitative evidence is also now being used within decision support tools such as the DECIDE evidence-to-decision frameworks [4] and SURE evidence-based policy briefs [14] and to inform decisions on implementation strategies. This wider use of qualitative evidence, including by organizations such as the World Health Organization (WHO), the European Commission Initiative on Breast Cancer and the National Institute for Health and Care Excellence (NICE) in the UK, has highlighted the need for the development of approaches that help users in deciding how much emphasis to give to such evidence in their decisions [15]. However, prior to the development of the approach described in this paper, there was no accepted, structured method for assessing confidence in the evidence from qualitative evidence syntheses

		[16]. The lack of such methods constrains the use of qualitative evidence to inform decision making.”
	'Aims of the CERQual approach'<br>* To what end would systematising and making these assessments of confidence more explicit improve this area? Is there something wrong with intuitively or informally assessing confidence of findings? What is the problem that producing the CERQual approach will address? Is it hoped that this approach will improve the quality of reviews and their reporting (i.e., the research side of things) or will it improve users' assessments of the findings (i.e., the user/decision-making side of things)? Or both?	As we see it, there are two main concerns with intuitively or informally assessing confidence in findings from qualitative evidence syntheses. Firstly, such assessments are not transparent – it is not possible for others to see how the assessments were made and decide whether they agree with the assessment decisions made. Secondly, different assessors may use different criteria for assessing confidence and so assessments are not systematised across assessors (or even from one assessment to another, for the same assessor). Combined with the lack of transparency, this makes it difficult to understand, and where necessary critique, the basis for assessments.<br><br>These concerns underlie the following statement in the paper “Broadly speaking, CERQual seeks to systematise these processes and make them explicit” (page 6). To make this point clearer, we have edited the paragraph as follows:<br>“The GRADE-CERQual approach (hereafter referred to as CERQual) has been developed to support people using findings from qualitative evidence syntheses in decision making processes. CERQual allows the user to make a transparent assessment of how much confidence decision makers and other users can place in individual review findings from syntheses of qualitative evidence. Many involved in using the findings of qualitative evidence syntheses may already be making these assessments of confidence intuitively or informally. As we see it, there are two main concerns with this: firstly, such assessments are not transparent and it is therefore not possible for others to see how the assessments were made and decide whether they agree with these decisions. Secondly, different assessors may use different criteria for assessing confidence and so assessments are not systematised across assessors (or even from one assessment to another, for the same assessor). Combined with the lack of transparency, this makes it difficult to understand, and where necessary critique, the basis for assessments. Broadly speaking, CERQual seeks to systematise the process of assessing confidence in the evidence from qualitative evidence syntheses and make these assessments explicit and transparent.”<br><br>As we note on page 6, “The GRADE-CERQual approach (hereafter referred to as CERQual) has been

		developed to support people using findings from qualitative evidence syntheses in decision making processes". Supporting people to use findings from qualitative evidence syntheses in decision making is the key 'problem' that we hope CERQual will address. However, it also has a number of others strengths, which we have outlined in Table 1. As the reviewer notes, CERQual may also, in the medium to long term, improve the quality and reporting of qualitative evidence syntheses but doing so is not our main aim in developing the approach.
	'Assumptions underlying the development of CERQual'<br>* This is a very helpful section and clearly/succinctly outlines the authors' assumptions for the reader.	Thank you
	'Applying CERQual across types of qualitative data and synthesis methods'<br>* This section raises some very interesting questions for future research.	Thank you
	'How was the CERQual approach developed?'<br>* This section describes a comprehensive process of developing CERQual with many types of users and refers the reader elsewhere for additional details, if desired. Including this section is very helpful in this introductory paper.	Thank you
	'An overview of the CERQual approach to assessing confidence in the evidence'<br>* It would be helpful to add a short example to more concretely describe what is meant by '[…] different enough that it might change how the finding influences a decision about health, social care, or other interventions' (p. 10, including lns. 24-34). Grounding this explanation in a short but material example (real or hypothetical) would help readers gain a more tangible understanding.	Thank you for this suggestion. We have added the following example on pages 10-11:<br>"For instance, if a review finding suggests that a new social care intervention is very acceptable to most service users, and we have high confidence in this finding (indicating that it is highly likely that the finding is a reasonable representation of acceptability to service users), decision makers may assess that it is appropriate to recommend that the intervention be implemented, assuming that the desirable effects outweigh the undesirable effects for other decision criteria. However, if we have very low confidence in this finding, and it is therefore unclear whether the intervention is acceptable to most service users, decision makers may assess that it is not appropriate to recommend its implementation."

* It is recommended that 'at least one member of the team applying CERQual has experience in both primary qualitative research and qualitative evidence synthesis' (p. 11, lns 33-36). Further explanation is needed. Why is this recommended? Is there research to demonstrate that users with experience in only one of these areas are insufficiently prepared to apply this approach? Would it have to be one member who has experience in both areas or could it involve two members who each have experience in one area? Also, and perhaps most importantly, what exactly is meant by 'experience' - this seems vague and highly subjective. Finally, should readers interpret this recommendation to mean that if a group of users does not have a member with experience in both of these areas, then that group should not apply the CERQual approach to a review of qualitative research (or that if they do, the result will be of a lower quality)?	We meant this as a general point about the skills required within a review team planning to apply CERQual, and were not trying to suggest that one member of the team should have all of these skills rather than, for example having two members have each have experience in one area.<br><br>This suggestion is based on our own experience of applying CERQual as well as our experience of training people in using CERQual and supporting other review teams who are applying the approach. This experience suggests that people who have little or no experience in qualitative methods find it difficult to grasp the details of how to apply the approach, since they don't have the necessary underlying knowledge and experience. We don't see this as a remarkable point – it's simply because CERQual builds on a wide body of knowledge with which users need at least some familiarity or, to coin a phrase, users need to know how to walk before they can run. We of course cannot prescribe who can and cannot apply CERQual and so have removed the word 'recommend'. We also have not formally assessed whether those who do not have relevant understanding or experience apply CERQual less appropriately than those who do have such understanding or experience.<br><br>To capture these points, we have edited the text as follows (page 12):<br>"We noted earlier that the development of CERQual has been informed by the principles and methods underlying both primary qualitative research and qualitative evidence synthesis. Those applying CERQual should, in our experience, have a good understanding of both qualitative primary research methods and qualitative evidence synthesis methods to apply the approach appropriately."<br><br>We have replaced 'experience' with 'understanding', which we think is clearer, but don't feel it would be helpful to be more specific since those using CERQual come to the process with a range of different backgrounds, training and skills.
'Purpose and structure of this series of papers'<br>* This section provides a helpful overview of the series.	Thank you
**Reviewer #2:**	
General comments	Many thanks for your support.

	Thank you for the opportunity to review this paper. As a researcher who has published several qualitative evidence syntheses, I have heard of CERQual over the last few years but have never seen a formal description of the approach. With an increase in both the production and use of qualitative evidence syntheses across many areas of heath and healthcare, this series is timely. As the authors point out, the articles will potentially be of interest to a range of readers/ users. My comments are minor and mainly relate to making the concept accessible to a wide readership.	
	Specific comments:<br>1. Page 5 line 5: Although the authors do mention it later on in the article, I think the fact that CERQual has been developed within health and healthcare (and that this is the context in which the article is written) and it has not yet been applied to other fields needs to be made clear at the beginning.	We have considered this suggestion, but do not think it would be appropriate to include this point at the start of the paper as the initial sections of the paper address broader issues, including why an approach to assessing confidence in the evidence from reviews of qualitative research is needed, the aims of the CERQual approach, the assumptions underlying the development of CERQual and applying CERQual across types of qualitative data and synthesis methods. We have, however, made in clearer in the section on 'How the CERQual approach was developed' that the initial development was within health and social care. As the reviewer mentions, we also note on page 13-14 of the paper that the current pool of worked examples is drawn largely from the areas of health and social care. Interestingly, though, examples from other fields are now starting to be published[1].
	2. Page 5 line 15: related to the above comment, the reference to institutions and systems is a bit abstract and needs context to make it meaningful; are the authors really referring to health systems and institutions?	We have tried to clarify this by providing some examples:<br>"This is particularly so for more complex interventions or policies as well as for programmes or policies whose implementation may impact across institutions and systems, such as across schools or across the education, health or social care system."
	3. Page 6 line 44: Again the authors do define this later on, but the term 'individual review finding' is first used on page 6 and to avoid confusion for readers unfamiliar with this, perhaps it warrants a definition here? The value of this series of papers partly lies in the authors' ability to	We have moved the definition of a review finding from page 11 to page 6.

[1] Day AM. Companion animals and natural disasters: A systematic review of literature. International journal of disaster risk reduction. 2017; 24: 81-90.

	make the concept accessible to a wide readership, many of whom will not know what an individual review finding is.	
	4. Page 7 line 1: Table 1 summarises strengths of the approach in a long list. Could the authors categorise these strengths somehow? I.e. strengths related to how the tool has been developed, those that relate to the process/conduct and those that relate to its various uses.	Thanks for this suggestion which we have implemented – please see page 21.
	5. Page 8 line 19: the applicability of CERQual to reviews that address different questions is discussed here. To make it really clear to a wide readership, the authors could provide further examples of the types of questions addressed by QES.	We have added the following sentence on page 8: "Within the domains of health and social care, this includes questions such as people's views or experiences of a health or social care issue, how different stakeholders and population groups value different health or social care outcomes, stakeholders' views on the acceptability and feasibility of health or social care interventions or options and on how an intervention might work, and factors affecting the implementation of an intervention or option."
	6. Pages 8-9: the process of developing CERQual, described here, seems to have been largely organic and unsystematic. Sub-headings might help the reader navigate this section better. Also its not particularly clear what a 'coffee CERQual' is, or why/how it is innovative.	The process of developing CERQual was necessarily organic as this was, to our knowledge, the first attempt to develop such an approach for qualitative evidence, and we did not have a development 'template' that we could follow. As we note in our earlier paper[2], the CERQual approach was initially developed to support a panel that was using qualitative evidence syntheses to develop a new World Health Organization (WHO) guideline and so the initial development of CERQual was in a context of a particular policy need. Our approach may have been unsystematic in the sense that it was not pre-specified, but we did include a range of key processes that we believe have resulted in a robust system. The organic nature of the development of CERQual, and the key processes that we used, are already described in this first paragraph of the methods section in this paper (page 9): "Overall we used a pragmatic and iterative approach to develop each CERQual component by brainstorming concepts within the development team, searching the literature for definitions, following up relevant citations, talking to experts in the field of qualitative evidence synthesis, developing consensus through multiple face-to-face meetings and teleconferences, and seeking feedback from ongoing engagement with the qualitative evidence synthesis community, the GRADE Working Group and organisations that commission, produce, or use

[2] Lewin S, Glenton C, Munthe-Kaas H, Carlsen B, Colvin CJ, Gülmezoglu M, et al. (2015) Using Qualitative Evidence in Decision Making for Health and Social Interventions: An Approach to Assess Confidence in Findings from Qualitative Evidence Syntheses (GRADE-CERQual). PLoS Med 12(10): e1001895.

		systematic reviews." We also note later in the methods section that, "While no formal guidelines exist for the development of an assessment approach of this kind, our process closely resembles the recommended approach for developing guidelines for reporting research processes". We have not made any changes to the text in this section but have added two subheadings, as suggested by the reviewer.<br><br>We agree that it is not clear what a 'coffee CERQual' is and have amended the text as follows (page 10): "Secondly, we implemented a small group feedback approach in which we facilitated brief discussions of individual CERQual components, either within our host organisations or in response to specific invitations from other organisations."
	7. Page 11 line 2: is CERQual applied in the same way, and indeed able to be applied, to the range of review findings mentioned here? i.e. are all four components equally applicable to a theory and a theme?	We believe that CERQual can be applied to the full range of review findings and that all four components will be applicable. However, we acknowledge on page 8-9 of the paper that "So far, experience in using CERQual has been concentrated in reviews with findings that are aggregative in nature; for example related to health care users' and providers' experiences and understanding of health issues and health service delivery. We have yet to gather experience about the use of CERQual on the full scope of synthesis methods and types of review findings. This is an important area for future research." Additional file 1 in paper 2 in this series provides guidance, based on what we know at this time, on deciding whether to apply CERQual to interpretive or explanatory level findings. We also acknowledge in paper 2 that we have limited knowledge and experience in this area. Further experience will allow us to develop more detailed guidance on this question.
	8. Page 13: the authors could elaborate here - especially for readers outside of the health field - is there any reason why CERQual couldn't be applied to reviews conducted in the fields of education, environment, agriculture etc?	We agree that CERQual could be applied to review findings from all fields, and have amended the text as follows (page 14): "However, because CERQual is a relatively new approach, the pool of worked examples is not yet extensive and is drawn largely from the areas of health and social care. However, we believe that CERQual can and should be applied to findings from qualitative evidence syntheses across all fields, including agriculture, crime and justice, education, the environment and international development, and we encourage readers to share with us their applied examples from these domains."
	9. Page 14: the reader must accept the authors' word that the approach has been well received, unless these	Good point, and we have now deleted this sentence.

experiences have been documented or written up elsewhere?	

Resubmission

23 Jul 2017 | Submitted | Manuscript version 2

9 Oct 2017 | Author responded | Author comments - Simon Lewin

General comments from the series editor	Author responses and changes made
Thanks for providing more methodological detail in the overview and subsequent papers. There are still some areas where it would be better if you could provide further details to reflect the amount of international developmental work undertaken e.g. databases searched, timeframes, how literature reviewed etc.	We have added further detail to the overall methods description in paper 1 of the series. Specifically, we have:<br>- Included the years during which we ran workshops and seminars to obtain feedback on CERQual, and the numbers of workshops and presentations undertaken<br>- Specified the period during which small group feedback sessions were run<br>- Specified the number of CERQual users and Project Group members interviewed<br><br>In the component papers (papers 3-6), we have noted that the literature searches that we undertook were informal in nature, as follows (example from paper 5):<br>"When developing CERQual's adequacy component, we undertook informal searches of the literature, including Google and Google Scholar, for definitions and discussion papers related to the concept of adequacy and to related concepts such as data quantity, sample size and data saturation."<br><br>We have also elaborated on the methods used to develop the content of paper 7 – please see below.
Ethics statements. Papers state that no humans were involved. Suggest amending to reflect consensus approach, interviews and questionnaires undertaken.	As we did not undertake formal data collection with people – all data collection was informal, in the context of training workshops, presentations and assessments of use of the approach, we have changed the ethics approval and consent to participate statements to the following:<br>"Not applicable. This study did not undertake any formal data collection involving humans or animals."
Titles and papers could reflect paper nth of # part in a series.	We have changed all titles to the following format, as agreed earlier (example from paper 1):<br>'Applying GRADE-CERQual to qualitative evidence synthesis findings – paper 1 of 7: Introduction to the series'

	State of the art has been removed from paper 6 but not all of the other papers in the series.	'State of the art' has been removed from all papers in the series.
	The new figure outlining the process is a good addition. As a reader I would have found it easier to read papers 3-6/7 before reading paper 2.	As discussed by email with Liz Glidewell, we had a very long debate within the group about this and concluded that there is no perfect order because paper 2 (overall assessment) and papers 3-6 (components) need to be seen together. We placed 'overall assessment' before the component papers as we felt that readers needed to understand what they were working towards before understanding each component. We feel that it would be best to keep the order as it is, but have made the following changes to assist readers:<br><br><u>Papers 2, 3, 4, 5 and 6:</u><br>We have inserted text along the lines of the following (example from paper 2 (p6): 'These component papers are closely related to this paper on making an overall CERQual assessment of confidence and creating a Summary of Qualitative Findings table. We have placed this paper before the four CERQual component papers as we think that it will be helpful for readers to understand how the component assessments will be used before discussing the details of how to apply each component.'<br><br><u>Papers 2, 3, 4, 5 and 6:</u><br>We have included in each paper an additional table that brings together all of the key definitions from each of the papers.
	Do you still want to publish paper 7 as a standalone or incorporate it into the overview along with the other ongoing research?	Yes, we feel that it works best as a standalone paper.
	Would the figure in the introduction outlining the process work better across all papers in the series as it contains more information than the figure just outlining the 4 and probable 5th component?	Thanks for this very helpful suggestion which we have implemented across all of the papers.
	**1. <u>Introduction</u>**	
	The lack of such methods constrains the use of…suggest reframing to "methods may constrain".	Change made
	"The CERQual approach is intended to be applied to well conducted syntheses." Could this be confusing to those applying the four components? Isn't CERQual designed to	We have not found this to be confusing in our interactions with users of CERQual. We feel that there would be little point in applying CERQual to a synthesis that has been poorly conducted as the findings of such

	provide evidence of confidence in a well conducted syntheses?	a synthesis are unlikely to be reliable and the synthesis is unlikely report transparently the methods used or to include sufficient information on the primary studies to allow a CERQual assessment to be undertaken. We take the same approach in relation to GRADE for effectiveness, for the same reasons. The problem is sometimes colloquially called 'garbage in-garbage out'!
	The section "Applying CERQual across types of qualitative data and syntheses methods". Would this be better placed after outlining how CERQual was developed?	We agree and have moved this section.
	"supported other teams". Can you say any more about the scale or settings involved?	We have provided more detail as follows:<br>"Thirdly, we applied the CERQual approach within diverse qualitative evidence syntheses in the areas of health and social care [6-8, 26-33] and also supported other teams in using CERQual by providing guidance through face-to-face or virtual training meetings and commenting on draft Summaries of Qualitative Findings tables. At least ten syntheses were supported in this way (for example, [34, 35])."
	Can you provided further detail about the questionnaire and qualitative interviews?	We have now provided further detail in the text and added an additional file listing the questions covered. The revised text reads as follows:<br>"We then gathered structured feedback from early users of CERQual through an online feedback form that was made available to all CERQual users and through short individual discussions with six members of review teams and two members of the CERQual Project Group. The questions included in the online feedback form and individual discussions are available in Additional File X."
	Summarise important areas for methodological research from table 4 in text for the readers ease?	We have revised the text as follows:<br>"Table 4 identifies several important areas for further methodological research, including how to apply CERQual in syntheses that include qualitative and quantitative data; how best to present CERQual assessments together with other kinds of evidence; ways of applying CERQual to syntheses of sources that have not used formal qualitative research procedures; and whether CERQual requires adaptation for application to more interpretive synthesis outputs, such as logic models."
	**2. <u>Making an overall assessment and summary of qualitative findings</u>**	
	Should the paragraph describing the four levels and rating down on p12 be moved to p10 under the 4 bulleted levels of concern?	This change has been made.

	Place the text relating to variation in assessors after the text outlining who should undertake an assessment?	This change has been made.
	Table 5. typo in component t missing.<br><br>Should you advise assessors to report how they've handled variation in levels of concern?	This typo has been corrected.
	**3. <u>Methodological limitations – problems design or conduct of primary studies</u>**	
	Consider adding a brief description of the Evidence Profile to p12.	Ok. We have now added the following parentheses describing the evidence profile on page 12 following the sentence: "Where you have concerns about methodological limitations, describe these concerns in the CERQual Evidence Profile in sufficient detail to allow users of the review findings to understand the reasons for the assessments made (The Evidence Profile presents each review finding along with an explanation of its CERQual assessment)"
	Link in text to table 2?	We have now added the following on page 9: "See Table 2 for an outline of areas where further work is needed with respect to critical appraisal tools for qualitative research."
	**4. <u>Coherence – How well finding supported by body of evidence 3500 3429</u>**	
	Consider adding a brief description of the Evidence Profile to p13.	We have added a brief description of the evidence profile on page 12:<br>"Where you have concerns about coherence, you should describe these concerns in the CERQual Evidence Profile in sufficient detail to allow users of the review findings to understand the reasons for the assessments made. The Evidence Profile presents each review finding along with the assessments for each CERQual component, the overall CERQual assessment for that finding and an explanation of this

	overall assessment. For more information, see the second paper in this series [19]."
**5. Adequacy of data – degree of richness and quantity of data 3500 2507**	
Consider contacting authors for further information as in other assessments?	We have added the following information to lines 204-205:<br><br>"An overview of the number of studies from which this data originated, and where possible, the number of participants or observations. Information about the number of participants or observations supporting each finding may be difficult to gain from the individual studies. While most studies describe the number of participants they included in their study overall or give some indication of the extent of their observations, they may be less clear about how well represented participants are in different themes and categories. You can contact study authors for additional information, but they may not be able to readily provide this level of detail. In these cases, this lack of information should be noted, and your assessment of data adequacy will have to be made based on the information available."
The sentence "For a description on descriptive and explanatory findings…" isn't embedded.	We have moved this sentence to lines 232-233.
Consider adding a brief description of the Evidence Profile to p12.	We have added the following information to lines 277-279:<br><br>The Evidence Profile presents each review finding along with the assessments for each CERQual component, the overall CERQual assessment for that finding and an explanation of this overall assessment.
**6. Relevance – extent applicable to context (perspective or population, phenomenon of interest, setting) of review question 3500 3551**	

I found a lot of the text more relevant to conducting a review than the CERQual assessment e.g. using theories and frameworks, how and when the review question should be developed, the pre-specification of sub-groups, strategies for identifying and selecting studies, trade-offs in searching.	Relevance is the only CERQual component that links directly to the review question. All the issues raised by the Editor need to be taken into consideration at the review design stage. We make this clear in the manuscript. See P6:<br><br>*'Relevance is the CERQual component that is anchored to the context specified in the review question. How the review question and objectives are expressed, how a priori subgroup analyses are specified, and how theoretical considerations inform the review design are therefore critical to making an assessment of relevance when applying CERQual.'*<br><br>See page 11: *'When assessing relevance, you should reflect on how the sample was located and on the underpinning principles that determined its selection….'*
Word missing p13 "You should if possible, that this"	Sincere apologies, this typo was corrected previously but the corrected draft was not uploaded last time.
Is it possible to comment on how the levels of concern map onto the different threats to relevance 'partial', 'indirect' and 'unclear'?	Tables 3, 4, 5 and 6 provide visual examples. Sincere apologies, these tables may not have been uploaded in error last time.
**7. <u>Dissemination bias – selective dissemination of studies or findings 2000 2455</u>**	
Methodological details e.g. 'consulting relevant literature' and 'additional empirical work'	We have added further detail as follows:<br><br>Abstract:<br>"We developed this paper by gathering feedback from relevant research communities, searching MEDLINE and Web of Science to identify and characterize the existing literature discussing or assessing dissemination bias in qualitative research and its wider implications, developing consensus through project group meetings, and conducting an online survey of on the extent, awareness and perceptions of dissemination bias in qualitative research."<br><br>Main text:

			"We used a pragmatic approach to develop our ideas on dissemination bias by consulting the literature on this topic, including searching MEDLINE and Web of Science to identify and characterize the existing literature discussing or assessing dissemination bias in qualitative research and its wider implications [3]; talking to experts in dissemination bias and qualitative evidence synthesis in a number of workshops; and developing consensus through multiple face-to-face CERQual Project Group meetings and teleconferences. We also undertook an online survey of researchers, journal editors and peer reviewers within the qualitative research domain on the extent, awareness and perceptions of dissemination bias in qualitative research [4]."
**Resubmission 2**			
9 Oct 2017	Submitted	Manuscript version 3	
**Publishing**			
17 Oct 2017	Editorially accepted		

How does Open Peer Review work?

Open peer review is a system where authors know who the reviewers are, and the reviewers know who the authors are. If the manuscript is accepted, the named reviewer reports are published alongside the article. Pre-publication versions of the article and author comments to reviewers are available by contacting info@biomedcentral.com. All previous versions of the manuscript and all author responses to the reviewers are also available.

You can find further information about the peer review system here.
